# Predictive capacity of obesity indices for high blood pressure among southern Ethiopian adult population: a WHO STEPS survey

**DOI:** 10.1186/s12872-020-01686-9

**Published:** 2020-09-21

**Authors:** Befikadu Tariku Gutema, Adefris Chuka, Gistane Ayele, Nega Degefa Megersa, Muluken Bekele, Alazar Baharu, Mekdes Kondal Gurara

**Affiliations:** 1 School of Public Health, Arba Minch University, P.O.Box 21, Arba Minch, Ethiopia; 2 Arba Minch Health and Demographic Surveillance System (HDSS), Arba Minch, Ethiopia; 3 Save the children international, Konso field Office, Knoso, Ethiopia; 4 Arba Minch University, School of Nursing, Arba Minch, Ethiopia; 5 Department of Computer Science, Arba Minch University, Arba Minch, Ethiopia

**Keywords:** Hypertension, obesity indices, cut-off values, Body Mass Index, Waist Circumference, Waist to Hip Ratio, Waist to Height Ratio

## Abstract

**Background:**

World Health Organization (WHO) consultation experts recommend countries to have guidance to identify public health action points suitable for their country. The objective of the study was to evaluate different obesity indices to predict high blood pressure and its optimal cutoff values among the adult population.

**Method:**

A total of 3368 individuals age from 25 to 64 years were included in this study. Data was collected based on the WHO Stepwise approach. Body mass index (BMI), waist circumference (WstC), waist to hip ratio (WHpR) and waist to height ratio (WHtR) were measured and calculated. High blood pressure was considered for those with systolic blood pressure above 135 mmHg, diastolic blood pressure above 85 mmHg or taking antihypertensive medications. To generate cutoff values, the receiver operator characteristic curve was generated with the maximum Youden index.

**Result:**

Women had a significantly higher hip circumference (*P =* 0.003), BMI (*P =* 0.036) and WHtR (*P <* 0.001) than men. Men had significantly higher WHpR (*P =* 0.027) than women. There were significantly higher BMI, WstC, WHpR, and WHtR among those with high blood pressure. The cutoff values for BMI, WstC, WHpR and WHtR were 22.86 kg/m^2^, 84.05 cm, 0.91 and 0.50 for men and 24.02 kg/m^2^, 79.50 cm, 0.91 and 0.51 for women, respectively.

**Conclusion:**

BMI, WstC, WHpR, and WHtR are a useful predictor of high blood pressure among adults’ rural residents of southern Ethiopia. As the sensitivity for the cutoff values of most of indices were low, further surveys in different settings may need to be done before a conclusion can be drawn on whether or not to review the anthropometric cut offs for high blood pressure in Ethiopia.

## Background

Anthropometric parameters that assess body fat are broadly utilized to foretell the increased risk of chronic disease both at individual and population levels [1]. Body mass index (BMI) has been used by the World Health Organization (WHO) to define the risk of metabolic syndrome, especially the severity of overweight and obesity [2]. In addition to BMI, determinants of central adiposity like waist circumference (WstC) and waist-to-hip ratio (WHpR) were adopted as accurate predictors of these syndromes [3]. Recently literature started to include waist to height ratio (WHtR) as an additional anthropometric indicator for assessment of overweight and obesity-related syndromes including hypertension [4].

Hypertension is regarded as one of the major contributing factors for the global burden of diseases and a cardiovascular risk factor [5]. Physiologically, weight gain is the most common cause of hypertension [6]. Studies indicate that there is an association between anthropometric indicators and adverse cardiovascular and metabolic outcomes, including hypertension [6–10]. This relationship is also seen among studies conducted in Ethiopia [11, 12]. The most frequently used anthropometric indices are BMI and WstC [13].

WHO identified a cutoff value for these anthropometric indicators commonly based on the body fat level. The risks of certain diseases are notably higher in some populations than would be expected [2, 14]. Again, literature identified that the capacity of anthropometric indicators for predicting risk factors and diseases differ based on sex, ethnicity and other factors [2, 15–21]. WHO consultation experts also recommend countries to have guidance to identify public health action points suitable for their country [2]. An urgency was recognized to develop and share best practices, including affordable and effective community-based programs to screen and treat hypertension during a meeting held in African Union member states in Addis Ababa. During the meeting, it was stated that hypertension as one of the continent’s greatest health challenges after HIV/AIDS [22]. Anthropometric indicators may be an efficient strategy for the detection and control of high blood pressure mainly because these measures can be implemented without specialized technical apparatus and easy to set goals for interventions [23, 24]. Therefore, the objective of the study was to evaluate different obesity indices (BMI, WstC, WHpR, and WHtR) to predict high blood pressure and its related sex-specific cutoff values among the adult population of southern Ethiopia.

## Methods

### Participants

The study was carried at Arba Minch Health and Demographic Surveillance System (HDSS) which is located in Arba Minch Zuria district Southern Ethiopia. Arba Minch, the administrative town of the district, located 505 km south from the capital city, Addis Ababa. Arba Minch HDSS includes nine Kebeles (the lowest administrative unit of Ethiopia) of Arba Minch Zuria District. Eight of the nine HDSS Kebeles are rural and one is semi-urban.

A community-based cross-sectional survey was conducted from April to June 2017. The source population was adult residents (25–64 years old). Based on the 2016 Arba Minch HDSS site report, 24,800 (11,854 Male and 12,946 Female) eligible individuals, which was 33.5% of the total population, were included as source population. Pregnant mothers or women who have a history of recent delivery up to 8 weeks were excluded from the study [25].

The sample size was determined based on the WHO stepwise (STEPS) approach to surveillance of chronic non-communicable disease risk [25]. The estimated sample size for a sex-age group was 421 and the final sample size was 3368. The sampling frame was extracted from the Arba Minch HDSS database using sex, date of birth, individual and household identifications as extraction variables. A simple random sampling technique using Stata version 14 was implemented to select the study participants from the Arba Minch HDSS database.

### Data collection procedure and instruments

Data collection instruments were adapted from STEPS instruments. From three levels of STEPS approach, only step one and two were applied in this study. Interview and measurement were conducted at the participants’ dwelling. Step one of the STEPS instruments is the questionnaire-based, which was designed to obtain core data on socio-demographic information with appropriate modifications in accordance with the STEPS manual [25]. Blood pressure measurements were taken using an Omron T9P digital automatic blood pressure monitor. Three blood pressure readings were taken on the left upper arm with the participant in a seated position following at least 15 min of rest. The participants rested for three minutes between each of the readings. The mean value of the three measurements was used as a final measurement of the blood pressure. Body weight (to the nearest 0.5 kg) was taken with the participant in bare feet with light clothing using SECA digital scale (model number 877). Height (to the nearest 1 cm) was measured using a stadiometer with participants wearing no shoes and without headwear. WstC measured at the midpoint between the palpable rib and the iliac crest. For measuring the hip circumference, the greatest posterior protuberance of the buttocks with a constant tension tape was used while the subject stands with arms at the sides, feet positioned close together, and weight evenly distributed across the feet.

### Data quality control

Training was given for three days on data collection material and measurement procedures for 20 data collectors and four supervisors. The pre-test was conducted on 2% of the sample size and the finding was used to adjust the content and approach of the tools. Supervisors had monitored the whole data collection process and checked the data for completeness every day during the data collection time. To increase the response rate, the data collectors repeatedly visited (at least three times) those participants who were not present at the house during data collection time.

### Definitions

BMI was generated by computing weight in kg per height in meter squared. WHpR and WHtR were computed by dividing the waist circumference by hip circumference and height, respectively. High blood pressure was considered for those with systolic blood pressure above 135 mmHg, diastolic blood pressure above 85 mmHg or if the participant reported that he/she is taking antihypertensive medications [26].

### Data processing and analysis

EPI-data version 3.1 statistical software was used for data entry and the data was exported to Stata version 14 for further management and analysis. Descriptive statistical analyses like mean, standard deviation, frequencies & percentages were computed. Pearson’s partial correlation coefficients were calculated to reflect the relationships between four obesity indexes, and to characterize how these indexes correlated with high blood pressure. The receiver operator characteristic (ROC) curve was generated to identify optimal cutoff values with the maximum Youden index (sensitivity plus specificity-1) for anthropometric indexes to blood pressure measurements and to determine the ability of anthropometric variables to discriminate high blood pressure. The area under the curve (AUC) was calculated to compare the effectiveness of the different indexes and *P*-value < 0.05 was considered significant.

## Results

A total of 3345 adults (50.0% were men) participated in the study with a response rate of 99.3%. The mean (SD) age of the participants was 44.59 (11.17) years and there was no significant difference in age between both sexes (*P* = 0.283). In addition, there was no significant difference between men and women concerning WstC (*P =* 0.406), systolic blood pressure (*P =* 0.837) and diastolic blood pressure (*P =* 8.99). However, women had a significantly higher hip circumference (*P =* 0.003), BMI (*P =* 0.036) and WHtR (*P <* 0.001) than men. Men had significantly higher WHpR (*P =* 0.027) than women (Table 1).

**Table 1:** Characteristics of adult (25–64 years of age) residents of Arba Minch HDSS, Southern Ethiopia

Variable	Total	Male	Female	***P***-Value
Number of participants	3345 (100%)	1673 (50.01%)	1672 (49.99%)	
Age (year)	44.59 (11.17)	44.80 (11.07)	44.38 (11.27)	0.283
Weight (kg)	54.98 (9.26)	58.03 (8.66)	51.93 (8.82)	< 0.001
Height (cm)	159.74 (8.74)	164.58 (7.78)	154.90 (6.75)	< 0.001
WstC (cm)	79.11 (8.24)	78.99 (7.73)	79.23 (8.72)	0.406
Hip circumference (cm)	88.66 (7.85)	88.26 (7.07)	89.06 (8.53)	0.003
BMI (Kg/m^2^)	21.50 (2.95)	21.40 (2.66)	21.61 (3.21)	0.036
WHpR	0.89 (0.07)	0.90 (0.07)	0.89 (0.07)	0.027
WHtR	0.50 (0.05)	0.48 (0.05)	0.51 (0.06)	< 0.001
Systolic Blood Pressure (mmHg)	122.38 (19.29)	122.31 (17.70)	122.45 (20.75)	0.837
Diastolic Blood Pressure (mmHg)	76.26 (11.40)	76.29 (11.78)	76.24 (11.01)	0.899

*BMI* Body mass index, *WHpR* Waist to hip ratio, *WHtR* Waist to height ratio, *WstC* Waist circumference

Significantly higher BMI, WstC, WHpR and WHtR were observed among those with high blood pressure, systolic blood pressure above 135 mmHg and diastolic blood pressure above 85 mmHg for men and total participants (for all *P* values were less than 0.05). For women, there were significantly higher BMI, WstC, WHpR and WHtR among those with high blood pressure and diastolic blood pressure above 85 mmHg. Only significantly higher WHpR and WHtR was observed in systolic blood pressure above 135 mmHg in women (Table 2).

**Table 2:** BMI, WstC, WHpR and WHtR values according to blood pressure measurements by sex of adult (25–64 years of age) residents of Arba Minch HDSSS, Southern Ethiopia

Sex	Indicators		Freq. (%)	Mean (SD) of Anthropometric Indicators			
				BMI	WstC	WHpR	WHtR
Male	Systolic	> = 135	303 (18.1)	21.84 (3.09)	81.72 (9.58)	0.91 (0.09)	0.49 (0.06)
		< 135	1370 (81.9)	21.30 (2.55)	78.39 (7.13)	0.89 (0.07)	0.48 (0.04)
		*P-*Value		0.001	< 0.001	< 0.001	< 0.001
	Diastolic	> = 85	318 (19.0)	22.15 (3.13)	81.71 (9.35)	0.91 (0.08)	0.49 (0.05)
		< 85	1355 (81.0)	21.22 (2.51)	78.36 (7.16)	0.89 (0.07)	0.48 (0.04)
		*P-*Value		< 0.001	< 0.001	< 0.001	< 0.001
	High Blood Pressure	Yes	437 (26.1)	21.97 (3.02)	81.42 (9.12)	0.91 (0.08)	0.49 (0.05)
		No	1236 (73.9)	21.20 (2.49)	78.14 (6.98)	0.89 (0.07)	0.48 (0.04)
		*P-*Value		< 0.001	< 0.001	< 0.001	< 0.001
Female	Systolic	> = 135	334 (20.0)	21.65 (3.69)	79.99 (9.96)	0.90 (0.07)	0.52 (0.06)
		< 135	1338 (80.0)	21.60 (3.07)	79.04 (8.38)	0.89 (0.07)	0.51 (0.05)
		*P-*Value		0.812	0.074	0.034	0.001
	Diastolic	> = 85	346 (20.7)	22.34 (3.95)	81.47 (10.13)	0.90 (0.06)	0.53 (0.06)
		< 85	1326 (79.3)	21.42 (2.95)	78.65 (8.22)	0.89 (0.07)	0.51 (0.05)
		*P-*Value		< 0.001	< 0.001	0.023	< 0.001
	High Blood Pressure	Yes	477 (28.5)	22.12 (3.80)	81.07 (10.17)	0.90 (0.07)	0.53 (0.06)
		No	1195 (71.5)	21.41 (2.91)	78.50 (7.96)	0.89 (0.07)	0.51 (0.05)
		*P-*Value		< 0.001	< 0.001	0.009	< 0.001
Both	Systolic	> = 135	637 (19.0)	21.74 (3.41)	80.81 (9.81)	0.91 (0.08)	0.51 (0.06)
		< 135	2708 (81.0)	21.45 (2.82)	78.71 (7.77)	0.89 (0.07)	0.49 (0.05)
		*P-*Value		0.024	< 0.001	< 0.001	< 0.001
	Diastolic	> = 85	664 (19.9)	22.25 (3.58)	81.58 (9.76)	0.90 (0.07)	0.52 (0.06)
		< 85	2681 (80.1)	21.32 (2.74)	78.50 (7.70)	0.89 (0.07)	0.49 (0.05)
		*P-*Value		< 0.001	< 0.001	< 0.001	< 0.001
	High Blood Pressure	Yes	914 (27.3)	22.05 (3.44)	81.24 (9.68)	0.90 (0.08)	0.51 (0.06)
		No	2431 (72.7)	21.30 (2.71)	78.31 (7.48)	0.89 (0.07)	0.49 (0.05)
		*P-*Value		< 0.001	< 0.001	< 0.001	< 0.001

*BMI* Body mass index, *WHpR* Waist to hip ratio, *WHtR* Waist to height ratio, *WstC* Waist circumference

**Fig. 1:**
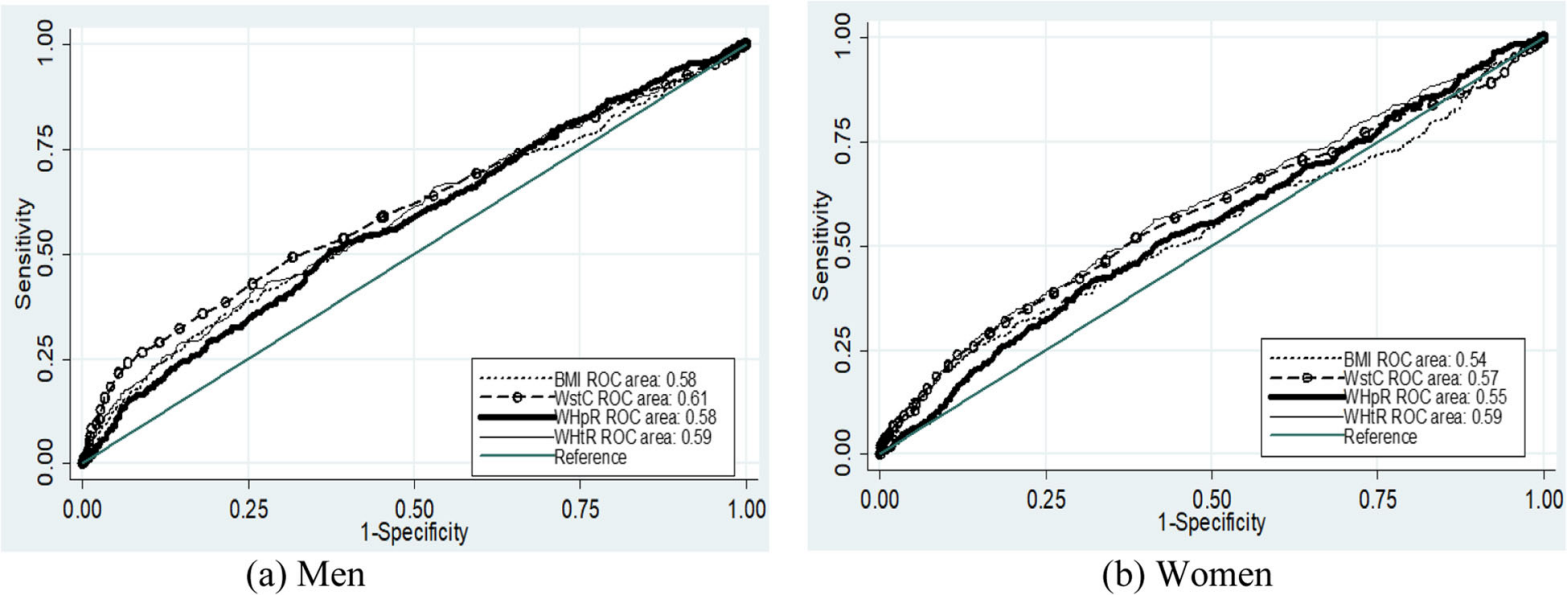
ROC curve for the assessment of the risk of high blood pressure on anthropometric indices

**Fig. 2:**
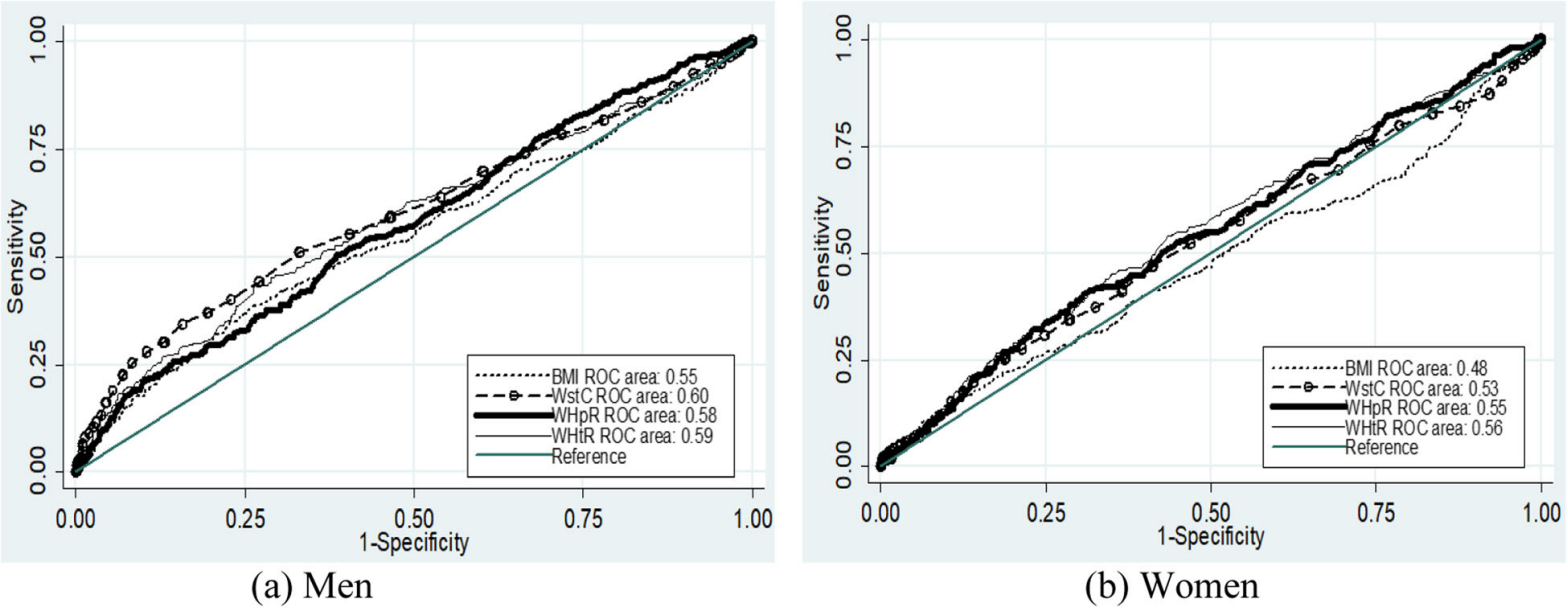
ROC curve for the assessment of the risk of systolic blood pressure over 135 mmHg on anthropometric indices

**Fig. 3:**
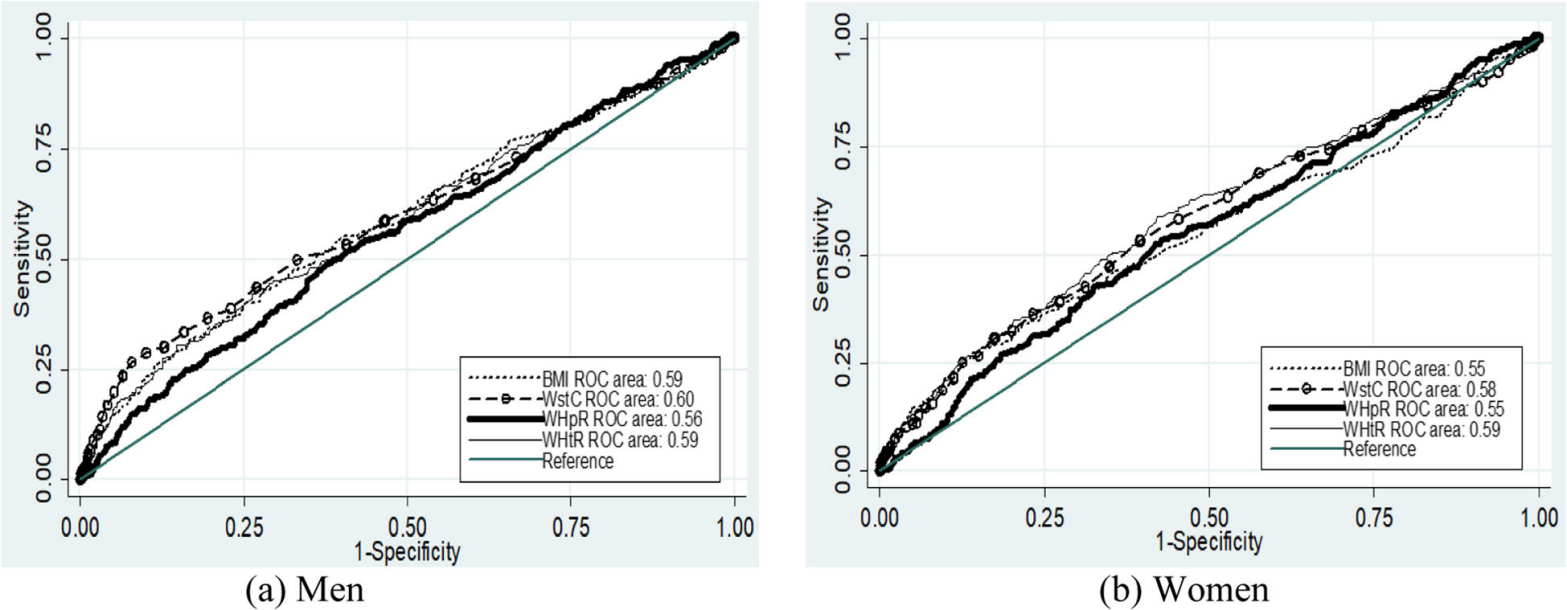
ROC curve for the assessment of the risk of diastolic blood pressure over 85 mmHg on anthropometric indices

Among men, WstC had the highest AUC followed by WHtR, WHpR and BMI for high blood pressure. All of the anthropometric indicators were significant for AUC for high blood pressure among men. For women, WHtR had a higher AUC and followed by WstC, WHpR and BMI for high blood pressure. Like men, all anthropometric indicators showed significant for AUC for high blood pressure for women. In the case of AUC for systolic blood pressure above 135 mmHg, WstC for men and WHtR for women were the highest AUC. The AUC was significant for all anthropometric indicators for men, whereas only WHtR and WHpR for women. In the case of diastolic blood pressure above 85 mmHg, WstC for men and WHtR for women had the highest AUC. All anthropometric indicators for diastolic blood pressure above 85 mmHg had a significant AUC for both men and women (Table 3 and Figs. 1, 2, and 3).

**Table 3:** AUC, optimal cutoff values, sensitivity and specificity of anthropometric indicators to predict high blood pressure according to sex of adult (25–64 years of age) residents of Arba Minch HDSSS, Southern Ethiopia

	Indices	Sex	AUC	Cutoff value	Sensitivity (%)	Specificity (%)	Youden index
High blood pressure	BMI	M	0.58 (0.54–0.61)	22.86	35.7%	78.8%	0.15
		F	0.54 (0.51–0.57)	24.02	26.4%	85.4%	0.12
	WstC	M	0.61 (0.58–0.64)	84.05	32.5%	85.4%	0.18
		F	0.57 (0.54–0.61)	79.50	52.0%	61.5%	0.13
	WHpR	M	0.58 (0.54–0.61)	0.91	50.8%	62.7%	0.14
		F	0.55 (0.52–0.59)	0.91	41.7%	68.2%	0.10
	WHtR	M	0.59 (0.56–0.62)	0.50	41.9%	74.0%	0.16
		F	0.59 (0.56–0.62)	0.51	56.4%	58.7%	0.15
Systolic blood pressure > 135 mmHg	BMI	M	0.55 (0.51–0.59)	22.57	38.6%	74.2%	0.13
		F	0.48 (0.44–0.52)	25.19	15.9%	88.9%	0.05
	WstC	M	0.60 (0.57–0.64)	84.05	34.7%	84.2%	0.20
		F	0.53 (0.49–0.56)	85.00	25.7%	81.0%	0.07
	WHpR	M	0.58 (0.54–0.61)	0.91	50.5%	61.3%	0.12
		F	0.55 (0.51–0.58)	0.91	40.4%	69.4%	0.10
	WHtR	M	0.59 (0.55–0.63)	0.50	43.6%	73.5%	0.17
		F	0.56 (0.52–0.59)	0.51	54.2%	56.6%	0.11
Diastolic blood pressure > 85 mmHg	BMI	M	0.59 (0.55–0.63)	21.53	54.4%	60.8%	0.15
		F	0.55 (0.52–0.59)	24.22	27.5%	86.1%	0.14
	WstC	M	0.60 (0.56–0.64)	87.00	26.7%	92.2%	0.19
		F	0.58 (0.55–0.62)	79.50	53.5%	60.6%	0.14
	WHpR	M	0.56 (0.53–0.60)	0.91	50.0%	61.3%	0.11
		F	0.55 (0.52–0.59)	0.90	52.6%	58.2%	0.11
	WHtR	M	0.59 (0.55–0.62)	0.52	30.8%	85.2%	0.16
		F	0.59 (0.56–0.63)	0.51	59.0%	57.9%	0.17

*AUC* Area Under the Curve, *BMI* Body mass index, *WHpR* Waist to hip ratio, *WHtR* Waist to height ratio, *WstC* Waist circumference

For high blood pressure among male, the cutoff value for BMI was 22.86 kg/m^2^, with 35.7% sensitivity and 78.8% specificity at maximum Youden index. For females, the cutoff values was 24.02 kg/m^2^ with 26.4% sensitivity and 85.4% specificity. The sensitivity and specificity for high blood pressure for the traditional cutoff value, which is 25 kg/m^2^, were 14 and 94% for men, and 20 and 90% for women, respectively. Regarding waist circumference, the cutoff values, for men and women, was 84 cm and 79.5 cm, respectively. The sensitivity and specificity were 32.5 and 85.4% for men and 52.2 and 61.5% for women, respectively. WHO cutoff values for waist circumference were 94 cm for men and 80 cm for women [27]. At these cutoff values, the sensitivity and specificity for men were nearly 10 and 98% and for women 52 and 62%, respectively. Based on the highest Youden index, the cutoff value for WHpR were 0.91 for both sexes with 50.8% sensitivity and 62.5% specificity for men and 52.0% sensitivity and 61.5% specificity for women. According to the WHO cutoff value for sustainably increased risk of metabolic complications was 0.90 and 0.85 for men and women, respectively [27]. At these cutoff values, the sensitivity and specificity for men were 54 and 58% and for women were 78 and 25%, respectively. Regarding the cutoff values for WHtR for men and women was 0.50 and 0.51, respectively. The specificity and sensitivity were 41.9 and 74.0% for men and 56.4 and 58.7% for women, respectively (Table 3 & Figs. 1, 2, 3).

## Discussion

This study showed that women had significantly higher mean BMI and WHtR compared to men. Men had significantly higher mean WHpR than women. Prior studies on the adult population in Ethiopia showed mean BMI ranges from 18.7 kg/m^2^ to 21.0 kg/m^2^, with higher among women [11, 28, 29], which is similar to the finding of this study. A cross-sectional community-based study among urban residents of Gondar, Northwest Ethiopia, showed that the mean WstC was 85.71 cm, with significantly higher among men participants [30]. A study in Gilgel Gibe Field Research Center, Southwest Ethiopia showed that the mean WstC was 75.2 cm and 73.8 cm for men and women, respectively, without significant difference [29]. Both studies conducted in the Gondor town and Gilgel Gibe Field Research Center are nearly similar mean WstC with this report. The report from Gilgel Gibe Field Research Center indicates that the mean WHpR was higher among men (0.90) compared to women (0.87) [29], which is similar to this finding regarding the difference between sex. Similarly, a study conducted at urban residents of Gondar was 0.89, with higher mean WHpR among men compared to women [30].

In this report, all the anthropometric indicators used (BMI, WstC, WHpR and WHtR) were apparently higher for high blood pressure. Similarly, a study conducted among 772 Chinese subjects showed that there was significantly higher mean value of BMI, WstC and WHtR for increased measurements of blood pressure. Contrary to the finding of this report, WHpR was not related [31]^.^ A study among rural Wardha, India showed BMI and waist circumference had a strong correlation with increased blood pressure [8]. A study among 2097 adult Nigerian also showed that there was an association between BMI and blood pressure, which was with higher BMI there was an increased risk of hypertension [32]. A report from three different HDSS including from Ethiopia indicated that BMI was significantly correlated with both systolic blood pressure and diastolic blood pressure for the Ethiopian adult population [11]. As it is indicated in different literature, the association between obesity and hypertension is related to insulin resistance, sodium retention, increased sympathetic nervous system activity, activation of renin–angiotensin–aldosterone, and altered vascular function [33–36].

This study indicated that BMI, WstC, WHpR and WHtR useful indicators to identify the presence of high blood pressure in the adult population of the study setting. In addition, except BMI and WstC for systolic blood pressure above 135 mmHg for women, the other indicators showed that they had useful cutoff values for increased blood pressure measurements. Different studies showed the importance of these anthropometric indicators as a predictor of high blood pressure [31, 37, 38].

The cutoff value recommended by the WHO for BMI is 25 kg/m^2^. The present study revealed that 22.86 kg/m^2^ and 24.02 kg/m^2^ were the cutoff values for indicating the presence of high blood pressure for men and women, respectively. Other Studies in Ethiopia and Asian countries showed similarly lower cutoff value than indicated by WHO based on the Caucasian population [27]. For men, the cutoff value for the Chinese Liaoning Province, Hong Kong, and Western Ethiopia were 23.0 kg/m^2^, 23.8 kg/m^2^ and 23.5 kg/m^2^, respectively, which are nearly similar to this finding. Similarly, for women, the cutoff values were 23.3 kg/m^2^, 24.1 kg/m^2^ and 26.2 kg/m^2^ for the Chines population of Liaoning Province, Hong Kong and Western Ethiopia, respectively [31, 37, 39]^.^ A study from Taiwanese and Shandong of China showed that the cutoff value of BMI was 25.74 kg/m^2^ and 25 kg/m^2^ for men and 23.46 kg/m^2^ and 24.5 kg/m^2^ for women, respectively [38, 40]. Finding from employees of Jimma University, Western Ethiopia showed a slightly higher BMI cutoff value than indicated for women by WHO based on the Caucasian population [27]. The higher cutoff values compared to WHO cutoff values for overweight might be related to urban society, which is the affiliated population in the case of Ethiopia. Studies conducted for assessing BMI cutoff values for determining high blood pressure have higher sensitivity and specificity than this finding for both men and women [31, 38–40].

The cutoff value for WstC to indicate high blood pressure of this study was lower than the cutoff value of WHO. The cutoff value by WHO is 94 cm for men and 80 cm for women [27]. A study on Taiwanese, Hong Kong, Shandong China and Western Ethiopia adults showed the cutoff value for WstC to predict high blood pressure was 87.9 cm, 82 cm, 88.5 cm and 89.2 cm for men and 76.4 cm, 78.4 cm, 83.5 cm and 93 cm for women [37–40]^.^ The cutoff value for women WstC was similar to the WHO cutoff recommendation. For men, the cutoff value is lower than the recommended. Relatively the specificity for both men and women, and sensitivity for women is good, the sensitivity for the WstC cut-off value for men was by far lower even compared to other studies [31, 38–40].

The cutoff value to predict high blood pressure by using WHpR was 0.91 for both sexes from this finding. A cutoff value for WHpR as a predictor for high blood pressure in Southwest Ethiopia was 0.86 and 0.89 for men and women employees of the University [37]^.^ Other studies, like Hong Kong, showed 0.89 for men and 0.84 for women as a predictor for high blood pressure [39]. Abdominal obesity is further defined as WHpR above 0.90 for males and above 0.85 for females [27]. The WHO recommendation is nearly similar for men but lower for women compared with this study. The sensitivity for the cutoff value of WHpR was lower than a study conducted in Liaoning Province and Hong Kong, China. The specificity was better for the study conducted Liaoning Province of China and nearly similar to the study conducted in Hong Kong, China [31, 39].

In this study, the WHtR cutoff value of identifying individuals with high blood pressure was nearly 0.50 for both sexes. A study among Hong Kong showed 0.50 for men and 0.55 for women as a cutoff value for WHtR as a predictor for high blood pressure among adult males [39]^.^ A study among Taiwanese male adults showed that the cutoff value was 0.51 for men and 0.49 for women [40]. A study from Shandong of China showed that the cutoff value of WHtR was 0.53 for men and 0.52 for women [38]. A study among the employees of Jimma University, Western Ethiopia showed 0.47 for men and 0.51 for women as a cutoff value [37]^.^ The specificity for the cutoff value for WHtR of this study was nearly similar to different studies. But the sensitivity was low for both men and women [38–40].

For this analysis, we consider measurements of blood pressure which is a part of metabolic syndrome. The data were collected mostly from rural residents within a district. As it is recommended to use different cutoff values for anthropometric indicators of metabolic syndrome, this finding gives a significant contribution.

## Conclusions

In conclusion, BMI, WstC, WHpR and WHtR are useful predictors of high blood pressure among adults of rural residents of southern Ethiopia. This report found that the cutoff value of BMI for blood pressure measurements lower than that of the WHO’s recommendation for overweight based on the Caucasian population. But the sensitivity and specificity of BMI cutoff values for determining high blood pressure were low. Regarding WstC, the cutoff value is similar to the WHO cutoff value for women, but it is lower than the cutoff value of the men participants. In addition, the sensitivity for assessing high blood pressure using WstC was low for men. WHO recommended the cutoff value for WHpR was similar for men but lower for the women population of the study site. For most of the anthropometric indicators, the cutoff value as a predictor of high blood pressure was lower in this population compared with the international cutoff values. In addition, the sensitivity for determining the cutoff values for most of the indices was low. Further surveys in different settings may need to be done before a conclusion can be drawn on whether or not to review the anthropometric cutoffs for high blood pressure in Ethiopia.

## Supplementary information

**Additional file 1.**

## Data Availability

The datasets used and analyzed during the current study is available from Arba Minch Demographic Surveillance and Health Research Center, Arba Minch University and corresponding author on reasonable request. The survey tool/questionnaire are available as supplementary information files.

## Abbreviations

AUC: Area under the curve
BMI: Body mass index;
HDSS: Health and demographic surveillance system
ROC: Receiver operator characteristic
STEPS: WHO stepwise
WHO: World Health Organization
WHpR: Waist to hip ratio
WHtR: Waist to height ratio
WstC: Waist circumference
