# Supplementary material for: Predictive capacity of obesity indices for high blood pressure among southern Ethiopian adult population: a WHO STEPS survey
Source: BMC Cardiovasc Disord. 2020 Sep 21;20:421. doi: 10.1186/s12872-020-01686-9 (PMC7507659; doi:10.1186/s12872-020-01686-9)
Supplement: Supplementary file 1 [file 12872_2020_1686_MOESM1_ESM.odt]

Title of the Research Project: Noncommunicable diseases and their risk factors survey using WHO STEPS system at Arba Minch Health and Demographic Surveillance Site (HDSS) 2017

Name of the Organization: Arba Minch University Department of Public Health College of Medicine 

Introduction:

Hello Dear Sir/Madam! 

My name is ------------------------, I am representing the study team coordinated by Arba Minch HDSS, at the Arba Minch University. I am collecting data on the study entitled: Noncommunicable diseases and their risk factors survey using WHO STEPS system at Arba Minch Health and Demographic Surveillance Site (HDSS) 2017

Purpose of the Research Project: The aim of the survey is to assess chronic disease risk factors at Arba Minch HDSS.

Procedure: The questionnaire has two components; Face-to-face interviews and physical measurements including measurement of blood pressure, waist circumference, hip circumference, weight and height will be done

Risk and /or Discomfort: The study does not have any risks to you. 

Benefits: There is no direct benefit to you for participating; however, the information obtained from the study may benefit the people of our country through using it for the design of programs to reduce the risk factors.

Confidentiality: Hence, your participation in this study is highly beneficial in order to generate information on the aforementioned issues. Any information you provide will remain confidential. Your name will not be identified in relation to your personal information. You will be informed of your measurements. 

Right to Refusal or Withdraw: If you don’t feel comfortable during the interview, or physical measurements you are free to discontinue your participation.

Person to contact: For unclear questions, you are kindly requested to ask the interviewer for clarity.

Consent: Did you understand the aim of the study? Yes ( )   No ( ).

 Are you willing to participate in the study? Yes ( )    No  ( ).

| Demographic Information |
| --- |
| Code | Question | Response | Skip |
| D01 | Participant house ID Number |  |  |
| D02 | Participant name and ID Number | Name ___________________ ID numbers ______________ |  |
| D03 | Date of completion of the instrument | ______________(DD/MM/YY) |  |
| D04 | Time of interview |  |  |
| D05 | Sex (Record Male / Female as observed) | 1. Male     2.Female |  |
| D06 | How old are you? | Years _______ |  |
| D07 | What is your educational status? | i. Illiterate  ii. Read and write  iii. Formal education  If formal education, highest educational level ____ |  |
| D08 | What is your ethnic background? | 1.      Gamo 2.      Zeyise 3.      Goffa 4.      Wolayita 5.      Amhara  6.      Others specify____ |  |
| D09 | What is your marital status? | 1. Never married 2. Currently married 3. Divorced 4. Widowed 5. Separated |  |
| D10 | What is your Religion? | 1. Protestant 2. Orthodox 3. Catholic 4. Muslim 5. Others, specify___ |  |
| D11 | Which of the following best describes your main work status over the past 12 months? | 1. Government employee 2. Non-government employee 3. Merchant 4. Student 5. Housewife 6. Retired 7. Farmer 8. Unemployed |  |
| D12 | How many people age 25-64 years, including yourself, live in your household? | __________Number of people |  |

| Step 1  Behavioral Measurements |
| --- |
| Tobacco Use |
| Now I am going to ask you some questions about tobacco use. |
| Code | Question | Response | Skip |
| T01 | Do you currently smoke any tobacco products, such as cigarettes, cigars or pipes or Gaya? | 1. Yes  2. No | T02 T08 |
| T02 | Do you currently smoke tobacco products daily? | 1. Yes  2. No |  |
| T03 | How old were you when you first started smoking? | Age (years) _______________ Don’t know 77 | T05 T04 |
| T04 | Do you remember how long ago it was? (RECORD ONLY 1, NOT ALL 3) Don’t know 77 | ____________ In Years OR ____________In Months OR ____________ In Weeks |  |
| T05 | On average, how many of the following products do you smoke each day/week? (IF LESS THAN DAILY, RECORD WEEKLY) | DAILY↓    WEEKLY↓  Manufactured cigarettes        ____        ____ Hand-rolled cigarettes           ____        ____ Pipes full of tobacco (Gaya)  ____        ____ Other                                      ____        ____ Other (please specify):__________________ |  |
| T06 | During the past 12 months, have you tried to stop smoking? | 1. Yes 2. No |  |
| T07 | During the past 12 months, did you advised by a doctor to stop smoking? | 1. Yes 2. No  3. I didn’t visit a doctor in past 12 month |  |
| T08 | In the past, did you ever smoke any tobacco products? (USE SHOWCARD) | 1. Yes 2. No | T09 T12 |
| T09 | In the past, did you ever smoke daily? | 1. Yes 2. No |  |
| T10 | How old were you when you stopped smoking daily? | Age (years) Don’t Know 77 | T27 T11 |
| T11 | How long ago did you stop smoking? (RECORD ONLY 1, NOT ALL 3) Don’t Know 77 | ______ Years ago OR     ______ Months ago OR ______ Weeks ago |  |
| T12 | Do you currently use any smokeless tobacco products such as [snuff, chewing tobacco, betel]?  (USE SHOWCARD) | 1. Yes  2. No | T13 T15 |
| T13 | Do you currently use smokeless tobacco products daily? | 1. Yes  2. No | T14 T15 |
| T14 | On average, how many times a day/week do you use ….  (IF LESS THAN DAILY, RECORD WEEKLY) | 1. Snuff (suret), by nose          Daily________   weekly ___________ 2. Chewing tobacco (magado)          Daily _________  weekly _________ 3. Other (please specify          Daily _________  weekly _________ |  |
| T15 | In the past, did you ever use smokeless tobacco products such as [snuff, chewing tobacco, or betel] | 1. Yes  2. No | T15 T17 |
| T16 | In the past, did you ever use smokeless tobacco products such as [snuff, chewing tobacco, or betel] daily? | 1. Yes  2. No |  |
| T17 | During the past 30 days, did someone smoke in your home? | 1. Yes  2. No |  |

| Alcohol Consumption |
| --- |
| The next questions ask about the consumption of alcohol. |
| Code | Question | Response | Skip |
| A01 | Have you ever consumed any alcohol such as beer, wine, spirits or locally ‘Araque’,’Tej’, ‘Tella’ or ‘Sew, cheqa [add other? (USE SHOWCARD OR SHOW EXAMPLES) | 1. Yes 2. No | A02 DE01 |
| A02 | Have you consumed any alcohol within the past 12 months? | 1. Yes 2. No |  |
| A03 | During the past 12 months, how frequently have you had at least one alcoholic drink? (READ RESPONSES, USE SHOWCARD) | Daily 5-6 days per week 3-4 days per week 1-2 days per week 1-3 days per month 2-4 days per month |  |
| A04 | Have you consumed any alcohol within the past 30 days? | 1. Yes 2. No | A05 DE01 |
| A05 | During the past 30 days, on how many occasions did you have at least one standard alcoholic drink? | Number  Don't know 77 |  |
| A06 | During the past 30 days, when you drank alcohol, how many standard drinks on average did you have during one drinking occasion? (USE SHOWCARD) | 1. Tella (Number) __________ 2. Teji (Number) ___________ 3. Beer (Number) __________ 4. Wine (Number) __________ 5. Areqae (Number) ________ 6. Cheka (Number) _________ 7. Other specify ___________ |  |
| A07 | During the past 30 days, what was the largest number of standard drinks you had on a single occasion, counting all types of alcoholic drinks together? | Largest number _________ Don't Know 77 |  |
| A08 | During the past 30 days, how many times did you have Six or more standard drinks in a single drinking occasion? | Number of times Don't Know 77 |  |
| A09 | During the past 7 days, did you consume any homebrewed alcohol? | 1. Yes 2. No | A10 DE01 |
| A10 | On average, how many standard drinks of the following did you consume during the past 7 days? | Homebrewed Chaqa ________ Homebrewed Tella ________ Home brewed Araqae _______ Home brewed Teji________ Other ________ (IF OTHERS SPECIFY) |  |

| Diet |
| --- |
| The next questions ask about the fruits and vegetables that you usually eat. I have a nutrition card here that shows you some examples of local fruits and vegetables. Each picture represents the size of a serving. As you answer these questions please think of a typical week in the last year. |
| Code | Question | Response | Code |
| DE01 | In a typical week, on how many days do you eat fruit? (USE SHOWCARD) | Number of days ____ Don't Know 77 | If Zero days, go to D03 |
| DE02 | How many servings of fruit do you eat on one of those days? (USE SHOWCARD) | Number of servings ____ Don't Know 77 |  |
| DE03 | In a typical week, on how many days do you eat vegetables? (USE SHOWCARD) | Number of days ____ Don't Know 77 | If Zero days, go to D05 |
| DE04 | How many servings of vegetables do you eat on one of those days? (USE SHOWCARD). | Number of servings ____ Don't Know 77 |  |
| DE05 | When do you add salt to your food during food cooking process? SELECT ONLY ONE) (USE SHOWCARD) | 1. After food cooked on the plate 2. At the end of food cooking but in the casserole/pot 3. At the middle of cooking food 4. At the beginning of cooking food |  |
| DE06 | How often do you think you add salt to your food to make it tasty? | 1. Always 2. Sometimes  3. Never |  |
| DE07 | What type of oil or fat is most often used for meal preparation in your household? (SELECT ONE or more) | 1. Vegetable oil 2. Lard or suet 3. Butter 4. We don’t use oil or fat this much 5. Never use oil or fat 6. Others (specify) 7. Don’t know 77 |  |

| CORE: Physical Activity |
| --- |
| Next I am going to ask you about the time you spend doing different types of physical activity in a typical week. Please answer these questions even if you do not consider yourself to be a physically active person. |
| Think first about the time you spend doing work. Think of work as the things that you have to do such as paid or unpaid work, study/training, household chores, harvesting food/crops, fishing or hunting for food, seeking employment. In answering the following questions 'vigorous-intensity activities' are activities that require hard physical effort and cause large increases in breathing or heart rate, 'moderate-intensity activities' are activities that require moderate physical effort and cause small increases in breathing or heart rate. |
| Code | Question | Response | Skip |
| P01 | Does your work involve vigorous-intensity activity that causes large increases in breathing or heart rate like [carrying or lifting heavy loads, digging or construction work] for at least 10 minutes continuously? [INSERT EXAMPLES]  (USE SHOWCARD. | 1. Yes 2. No | P02 P04 |
| P02 | In a typical week, on how many days do you do vigorous-intensity activities as part of your work? | Number of days ____ |  |
| P03 | How much time do you spend doing vigorous-intensity activities at work on a typical day? | ____     _____ Hours :Minutes |  |
| P04 | Does your work involve moderate-intensity activity, which causes small increases in breathing or heart rate such as brisk walking [or carrying light loads] for at least 10 minutes continuously? [INSERT EXAMPLES]  (USE SHOWCARD) | 1. Yes 2. No | P05 P07 |
| P05 | In a typical week, on how many days do you do moderate-intensity activities as part of your work? | Number of days____ |  |
| P06 | How much time do you spend doing moderate-intensity activities at work on a typical day? | ____     _____ Hours :Minutes |  |
| Travel to and from places |
| The next questions exclude the physical activities at work that you have already mentioned. |
| Now I would like to ask you about the usual way you travel to and from places. For example to work, for shopping, to market, to place of worship. [Insert other examples if needed] |
| P07 | Do you walk or use a bicycle (pedal cycle) for at least 10 minutes continuously to get to and from places? | 1. Yes 2. No |  |
| P08 | In a typical week, on how many days do you walk or bicycle for at least 10 minutes continuously to get to and from places? | Number of days____ |  |
| P09 | How much time do you spend walking or bicycling for travel on a typical day? | ____     _____ Hours :Minutes |  |
| Sedentary behavior |
| The following question is about sitting or reclining at work, at home, getting to and from places, or with friends including time spent sitting at a desk, sitting with friends, traveling in car, bus, train, reading, playing cards or watching television, but do not include time spent sleeping. |
| P10 | How much time do you usually spend sitting or reclining on a typical day? | ____     _____ Hours :Minutes |  |

| Khat Chewing |
| --- |
| The next questions ask about chewing Khat. Khat (Catha edulis) is a plant grown commonly in the horn of Africa. Its young buds and tender leaves are chewed to attain a state of euphoria and stimulation. |
| Code | Question | Response | Skip |
| K01 | Have you ever chewed khat?  (USE SHOWCARD OR SHOW EXAMPLES) | 1. Yes 2. No | K02 C01 |
| K02 | Have you chewed khat in the past 12 months? | 1. Yes 2. No | K03 C01 |
| K03 | During the past 12 months, how frequently have you had khat? (READ RESPONSES, USE SHOWCARD) | 1. Daily  2. 3-4x per Week 3. Weekly 4. 2-3x per months 5. Monthly  6. Very occasionally |  |
| K04 | Have you chewed khat within the past 30 days? | 1. Yes 2. No | K05 C01 |
| K05 | During the past 30 days, on how many occasions did you have khat? | Number ______ Don't know 77 |  |
| K06 | During the past 30 days, when you consumed khat, how often was it with cigarette smoking? | 1. Always 2. Sometimes 3. Rarely 4. Never |  |
| K07 | During the past 30 days, when you consumed khat, how often was it followed by alcohol drinking to break the effect of khat? | 1. Always 2. Sometimes 3. Rarely 4. Never 77. Don't Know |  |
| K08 | During the past 30 days, when you consumed khat, how often was it accompanied by consuming Shisha or other stimulants (Please mention if any)? | 1. Always 2. Sometimes 3. Rarely 4. Never 5. Other stimulants specify |  |

| Coffee Consumption |  |
| --- | --- |
|  |
| The next questions ask about the consumption of coffee |  |
| Code | Question | Response | Skip |  |
| C01 | Have you ever consumed any coffee or coffee leaf or tea? | 1. Yes 2. No | C02 M01 |  |
| C02 | On average, how often do you drink coffee, or tea ? (Please tick one option)? | 1. Daily 2. 2-3 times per day 3. >3 times per days 4. 1-2 times per week 5. > 3 times per week 6. Rarely in two weeks |  |  |
| C03 | On average, how often do you drink coffee leaf tea? (Please tick one option)? | 1. Daily 2. 2-3 times per day 3. >3 times per days 4. 1-2 times per week 5. > 3 times per week 6. Rarely in two weeks |  |  |
| C04 | What types of coffee you consumed today? | 1. Coffee 2. Coffee leaf tea 3. Both 4. I do not consumed |  |  |
| C05 | On average, how many cups of coffee drinks of the following do you consume daily? | Coffee    ________ Coffee leaf tea _____ |  |  |

| Meat  Consumption |
| --- |
| The next questions ask about the consumption of meat |
| Code | Question | Response | Skip |
| M01 | Have you ever consumed any meat product? | 1 Yes 2. No | Mo2 MS01 |
| M02 | On average, how often do you eat? (Please tick one option)?   Cooked meat any type  (beef, goat, sheep) | 1. Daily  2. 2-3 times per day  3. 1-2 times per week  4. > 3 times per week  5. Monthly  6. 2-4 times per year  7. Never |  |
| M03 | On average, how often do you eat Raw meat any type (beef, goat, and sheep)? | 1. Daily  2. 2-3 times per day  3. 1-2 times per week  4. > 3 times per week  5. Monthly  6. 2-4 times per year  7. Never |  |
| M04 | On average, how often do you eat Fish? | 1. Daily  2. 2-3 times per day  3. 1-2 times per week  4. > 3 times per week  5. Monthly  6. 2-4 times per year  7. Never |  |
| M05 | On average, how often do you eat Chicken? | 1. Daily  2. 2-3 times per day  3. 1-2 times per week  4. > 3 times per week  5. Monthly  6. 2-4 times per year  7. Never |  |
| M06 | When you ate Raw meat, how did it usually? | 1. Red   2. White   3. Both |  |
| M07 | When you ate cooked meat, how did it usually? | 1. Red   2. White   3. Both |  |

| Mental stress (Optional) Using SRQ 20 Instrument |  |
| --- | --- |
|  |
| Self-report questionnaire (SRQ20) |  |
|  |  |  |  |  |
| Code | Questions | Yes | No |  |
| MS01 | Do you often have Headaches? |  |  |  |
| MS02 | Is your appetite poor? |  |  |  |
| MS03 | Do you sleep badly? |  |  |  |
| MS04 | Are you easily frightened? |  |  |  |
| MS05 | Do your hands shake? |  |  |  |
| MS06 | Do you feel nervous, tense or worried? |  |  |  |
| MS07 | Is your digestion is poor? |  |  |  |
| MS08 | Do you have trouble thinking clearly? |  |  |  |
| MS09 | Do you feel un happy? |  |  |  |
| MS10 | Do you cry more than usual? |  |  |  |
| MS11 | Do you find it difficult to enjoy your daily activities? |  |  |  |
| MS12 | Do you find it difficult to make decisions? |  |  |  |
| MS13 | Is your daily work suffering? |  |  |  |
| MS14 | Are you unable to play a useful part in life? |  |  |  |
| MS15 | Have you lost interest in things? |  |  |  |
| MS16 | Do you feel that you are a worthless person? |  |  |  |
| MS17 | Has the thought of ending your life been in your mind? |  |  |  |
| MS18 | Do you feel tired all the time? |  |  |  |
| MS19 | Do you have un comfortable feelings in your stomach? |  |  |  |
| MS120 | Are you easily tired? |  |  |  |

| History of Hypertension |
| --- |
| Question | Response | Skip |
| HBP01 | Have you ever had your blood pressure measured by a doctor or other health worker and told by a doctor or other health worker that you have hypertension or hypertension? | 1. Yes 2. No | HBP02 HD01 |
| HBP02 | Have you been told in the past 12 months? | 1. Yes 2. No |  |
| HBP03 | Are you currently receiving Drugs (medication) that you have taken in the past two weeks for high blood pressure prescribed by a doctor or other health worker? | 1. Yes 2. No |  |
| HBP04 | Are you Advised by a doctor or other health worker to reduce salt intake for high blood pressure? | 1. Yes 2. No |  |
| HBP05 | Are you currently receiving Advice or treatment to lose weight and  start or do more exercise for high blood pressure prescribed by a doctor or other health worker? | 1. Yes 2. No |  |
| HBP06 | Are you currently receiving Advice or treatment by a doctor or other health worker to stop smoking? | 1. Yes 2. No |  |
| History of Diabetes |
| Question | Response | SKIP |
| HD01 | Have you ever had your blood sugar measured by a doctor or other health worker and told by a doctor or other health worker that you have raised blood sugar or diabetes?? | 1. Yes 2. No | HD02 W01 |
| HD02 | Have you been told in the past 12 months? | 1. Yes 2. No |  |
| HD03 | Are you currently receiving Drugs (medication) that you have taken in the past two weeks for diabetes prescribed by a doctor or other health worker? | 1. Yes 2. No |  |
| HD04 | Are you currently receiving Special prescribed diet for diabetes prescribed by a doctor or other health worker? | 1. Yes 2. No |  |
| HD05 | Are you currently receiving Advice or treatment to lose weight nd to start or do more exercise for diabetes prescribed by a doctor or other health worker? | 1. Yes 2. No |  |
| HD06 | Are you currently receiving Advice or treatment to stop smoking for diabetes prescribed by a doctor or other health worker? | 1. Yes 2. No |  |

| Current family wealth condition (wealth Index) |
| --- |
| S.No | Asset type | Response |  |
| W01 | Domestic Animal | Ox | 1-Yes | 2-No |  |
| Cow | 1-Yes | 2-No |  |
| Calf | 1-Yes | 2-No |  |
| Sheep | 1-Yes | 2-No |  |
| Goat | 1-Yes | 2-No |  |
| Horse | 1-Yes | 2-No |  |
| Donkey | 1-Yes | 2-No |  |
| Cock and Hen | 1-Yes | 2-No |  |
| W02 | Durable assets | Television | 1-Yes | 2-No |  |
| Radio | 1-Yes | 2-No |  |
| Electricity | 1-Yes | 2-No |  |
| Refrigerator | 1-Yes | 2-No |  |
| Conventional telephone | 1-Yes | 2-No |  |
| Mobile phone | 1-Yes | 2-No |  |
| Car | 1-Yes | 2-No |  |
| Motorcycle | 1-Yes | 2-No |  |
| Cycle | 1-Yes | 2-No |  |
| Cart | 1-Yes | 2-No |  |
| Gold ,money | 1-Yes | 2-No |  |
| W03 | house, land and productive asset | Ownership of Agricultural  land | 1-Yes | 2-No |  |
| Ownership of owned living house | 1-Yes | 2-No |  |
| Plough plow | 1-Yes | 2-No |  |
| Axe | 1-Yes | 2-No |  |
| Hoe | 1-Yes | 2-No |  |
| Shovel | 1-Yes | 2-No |  |
| Sickle | 1-Yes | 2-No |  |
| Modern beehive | 1-Yes | 2-No |  |
| Traditional beehive | 1-Yes | 2-No |  |
| W04 | Housing characteristic | Indoor plumping | 1-Yes | 2-No |  |
| Type of floor | 1-Cement/raw wood 2- Earth/dung |  |
| Toilet facility | 1- Sanitary/ improved pit latrine 2- Unsanitary /traditional |  |
| W05 | Other household material | Sofa | 1-Yes | 2-No |  |
| Bed | 1-Yes | 2-No |  |
| Table | 1-Yes | 2-No |  |
| Chair | 1-Yes | 2-No |  |
| Stove | 1-Yes | 2-No |  |

| House hold food security status |
| --- |
| Code | Question | Option |
| FS01 | In last 4 weeks, did you worried whether your food would run out before you got money to buy more? 1-Yes 2- No | If yes, how often did this happen? 1. One or two times in last four weeks 2. Three up to ten times in last four weeks 3. Greater than ten times in last four weeks |
| FS02 | In the last 4 weeks, did you/ your family members ever cut the size of your meals or skip meals because there wasn't enough food? 1. Yes      2. No | If yes, how often did this happen? 1. One or two times in last four weeks 2. Three up to ten times in last four weeks 3. Greater than ten times in last four weeks |
| FS03 | In last 4 weeks did you/your family members eat only the limited food because there was not enough food? 1- Yes   2- No | If yes, how often did this happen? 1. One or two times in last four weeks 2. Three up to ten times in last four weeks 3. Greater than ten times in last four weeks |
| FS04 | In last 4 weeks did you/your family members obligated to eat food that you/your family member did not want because here was not enough food? 1-Yes  2- No | If yes, how often did this happen? 1. One or two times in last four weeks 2. Three up to ten times in last four weeks 3. Greater than ten times in last four weeks |
| FS05 | In the last 4 weeks, did you/family member ever eat less than you felt sate because there wasn't enough food? 1-Yes  2- No | If yes, how often did this happen? 1. One or two times in last four weeks 2. Three up to ten times in last four weeks 3. Greater than ten times in last four weeks |
| FS06 | In the last 4 weeks, didn’t you get food ever because you/your family members just couldn't afford it? 1-Yes  2- No | If yes, how often did this happen? 1. One or two times in last four weeks 2. Three up to ten times in last four weeks 3. Greater than ten times in last four weeks |
| FS07 | In the last 4 weeks, were you ever hungry but didn't eat because you couldn't afford enough food? 1-Yes  2- No | If yes, how often did this happen? 1. One or two times in last four weeks 2. Three up to ten times in last four weeks 3. Greater than ten times in last four weeks |
| FS08 | In the last 4 weeks, did you/your family members ever not eat for a whole day because there wasn't enough money for food? 1-Yes  2- No | If yes, how often did this happen? 1. One or two times in last four weeks 2. Three up to ten times in last four weeks 3. Greater than ten times in last four weeks |
| FS09 | In the past 4 weeks, did you or any household member go a whole day and night without eating anything because there was not enough food? 1-Yes  2- No | If yes, how often did this happen? 1. One or two times in last four weeks 2. Three up to ten times in last four weeks 3. Greater than ten times in last four weeks |

| Step 2  Physical Measurements |
| --- |
| CORE: Blood Pressure |
| Code | Measurement | Record |  |  |
| BP01 | Reading 1 | Systolic ( mmHg) | └─┴─┴─┘ |  |  |
| Diastolic (mmHg) | └─┴─┴─┘ |  |  |
| BP02 | Reading 2 | Systolic (mmHg) | └─┴─┴─┘ |  |  |
| Diastolic (mmHg) | └─┴─┴─┘ |  |  |
| BP03 | Reading 3 | Systolic (mmHg) | └─┴─┴─┘ |  |  |
| Diastolic (mmHg) | └─┴─┴─┘ |  |  |
| CORE: Height and Weight and  Waist |
| HW01 | Height | in Centimetres (cm) | └─┴─┴─┘. └─┘ |  |  |
| HW02 | Weight | in Kilograms (kg) | └─┴─┴─┘.└─┘ |  |  |
| HW03 | Waist circumference | in Centimeters (cm) | └─┴─┴─┘.└─┘ |  |  |
| HW04 | Hip circumference | in Centimeters (cm) | └─┴─┴─┘.└─┘ |  |  |
